# Controllable Nucleation of Cavitation from Plasmonic Gold Nanoparticles for Enhancing High Intensity Focused Ultrasound Applications

**DOI:** 10.3791/58045

**Published:** 2018-10-05

**Authors:** James R. McLaughlan

**Affiliations:** ^1^School of Electronic and Electrical Engineering, University of Leeds; ^2^Leeds Institute of Cancer and Pathology, University of Leeds

**Keywords:** This Month in JoVE, Issue 140, High intensity focused ultrasound, photoacoustics, plasmonic nanoparticles, cavitation detection, inertial cavitation, thermal ablation, diagnostic ultrasound

## Abstract

In this study, plasmonic gold nanoparticles were simultaneously exposed to pulsed near-infrared laser light and high intensity focused ultrasound (HIFU) for the controllable nucleation of cavitation in tissue-mimicking gel phantoms. This *in vitro* protocol was developed to demonstrate the feasibility of this approach, for both enhancement of imaging and therapeutic applications for cancer. The same apparatus can be used for both imaging and therapeutic applications by varying the exposure duration of the HIFU system. For short duration exposures (10 µs), broadband acoustic emissions were generated through the controlled nucleation of inertial cavitation around the gold nanoparticles. These emissions provide direct localization of nanoparticles. For future applications, these particles may be functionalized with molecular-targeting antibodies (e.g. anti-HER2 for breast cancer) and can provide precise localization of cancerous regions, complementing routine diagnostic ultrasound imaging. For continuous wave (CW) exposures, the cavitation activity was used to increase the localized heating from the HIFU exposures resulting in larger thermal damage in the gel phantoms. The acoustic emissions generated from inertial cavitation activity during these CW exposures was monitored using a passive cavitation detection (PCD) system to provide feedback of cavitation activity. Increased localized heating was only achieved through the unique combination of nanoparticles, laser light and HIFU. Further validation of this technique in pre-clinical models of cancer is necessary.

**Figure:**
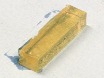


## Introduction

High intensity focused ultrasound (HIFU), or focused ultrasound surgery (FUS), is a non-ionizing and non-invasive technique that is used for the thermal ablation of subcutaneous tissue^1^. The main use of HIFU is in the treatment of soft tissue tumors^2^, but it is starting to be used for other applications, such as treating bone tumors^3^ or neurological conditions^4^. There are two main factors that limit the widespread use of HIFU in the clinic: firstly, difficulties in treatment guidance and secondly, long treatment times^5^. The combination of HIFU, pulsed laser illumination, and plasmonic gold nanorods described by this method could provide a way to overcome the current limitations for HIFU^6^.

During HIFU exposures, the dominant mechanism of tissue ablation is thermal damage. However, cavitation activity can also play a role^8^. Cavitation activity that occurs during HIFU exposures can consist of both mechanically and/or thermally mediated cavitation. Mechanically mediated cavitation is generally referred to as acoustic cavitation^7^, which is further subcategorized as bubbles undergoing either non-inertial or inertial^9^ behavior. Thermally mediated cavitation is from the formation of gas pockets, through ex-solution or vaporization, and is commonly referred to as 'boiling'^10^. Cavitation activity, most commonly inertial cavitation, has been shown to enhance the thermal heating rates achievable through HIFU exposures^11^ and thus help address one of its key limitations. However, the formation and activity of cavitation during HIFU exposures can be unpredictable and lead to negative effects such as over-treated regions, or asymmetrical thermal ablation^12^. In order to control cavitation activity during HIFU exposures, the introduction of external nuclei has been investigated. These can take the form of microbubbles^13^, phase-shift nanoemulsions^14^ or plasmonic nanoparticles^15^. Both microbubbles and nanoemulsions have been shown to improve signal-to-noise for imaging and enhanced thermal ablations. However, their transient nature means they have limited functionality over repeated HIFU exposures. Monitoring of cavitation activity during HIFU exposures is done using either active or passive cavitation detection (ACD or PCD, respectively). PCD is a favored technique for cavitation detection, as it can be performed concurrently with HIFU exposures and provides spectral content information. This spectral content can then be further analyzed to help identify the type of cavitation activity occurring^16^. Broadband acoustic emissions are used, since these emissions are unique to the presence of inertial cavitation^10^ and are linked to enhanced HIFU heating^11^.

Photoacoustic imaging (PAI) is an emerging clinical imaging technique^17^, which combines the spectral selectivity of pulsed laser excitation with the high resolution of ultrasound imaging^18^. It has previously been used to guide HIFU exposures^19^, but this imaging technique is limited by the penetration depth of laser light. Plasmonic gold nanoparticles can be used to act as 'contrast agents' increasing the local absorption of laser light and subsequently the amplitude of photoacoustic emissions^20^. For sufficiently high laser fluences, it is possible to cause the generation of microscopic vapor bubbles that can be used for highly localized imaging^21^. However, these exposure levels typically exceed the maximum permissible exposure limit for the use of laser light in humans^22^, and thus have limited use. The method employed in this study has previously shown that by simultaneously exposing the plasmonic nanoparticles to both laser illumination and HIFU, the laser fluence and acoustic pressures needed to nucleate these small vapor bubbles is dramatically reduced, and the signal-to-noise ratio for imaging is increased^23^. A method is described here for combining plasmonic nanoparticles with both laser and HIFU exposures for a highly controllable technique for the nucleation and activity of vapor bubbles.

## Protocol

### 1. Tissue Mimicking Phantom Manufacture

NOTE: An in-depth analysis of the acoustic properties of the optically transparent tissue-mimicking phantom used for all exposures in this study can be found in Choi,* et al.*^24^

NOTE: Each phantom mold contains approximately 50 mL of solution, and for each batch a total of five molds are filled. Thus, a total of 250 mL of phantom solution is prepared.

1. Add 148.2 mL (60% v/v) of deionized, filtered and degassed water to a 500 mL glass beaker and leave to equilibrate to room temperature. Add 75 mL of 40% (weight/volume) Acrylamide/Bis-acrylamide solution (30% v/v) to the glass beaker, followed by 25 mL of 1 M TRIS buffer, pH 8 (10% v/v), and 2.15 mL of 10% ammonium persulfate (APS; 0.86% v/v).
2. Place the glass beaker inside a vacuum chamber that is situated on a magnetic stirrer plate, and place a 40 mm long polytetrafluoroethylene (PTFE) magnetic stirring bar inside the beaker. With a medium stirring speed (*i.e., *ensure good mixing without for formation of vortex in the water), slowly add 22.5 g (9% w/v) of bovine serum albumin (BSA) powder.
3. Once all BSA has been added to the solution, close the vacuum chamber and turn on the vacuum pump. Maintain a vacuum of 80 mBar/H and continue stirring for a further 60 min, after which release the vacuum. At this point the solution should be clear with a slight yellow tint.
4. The above methodology is the same for phantoms made both with and without nanoparticles. If nanoparticles are required, add 10 µL (concentration of 1x10^8^ np/mL) of nanorods that have a surface plasmon resonance (SPR) at 850 nm and a diameter of 40 nm.
5. Finally, add 125 µL of tetramethylethylenediamine (TEMED) to catalyze polymerization of the phantom. Wait a further 5 min to allow for mixing, then pour the phantom solution into 5 individual molds and wait 20 min to set. Once set, remove them from the holders and store in an airtight container until use. Use phantoms within 24 h of manufacture.

### 2. Calibration of HIFU Transducers Free Field Acoustic Pressure

NOTE: This section of the protocol is not necessary before every lesioning/imaging experiment. It is a calibration procedure to be performed at regular intervals to ensure acoustic output of the system is correct.

1. Fill an acrylic water tank (280x141x132 mm) with 4.5 L of deionized and degassed water. Mount the HIFU transducer on a fixed position post at one end of the tank, facing in. Parallel to this, mount a calibrated (performed by the National Physical Laboratories) membrane hydrophone onto a three-axis manual micrometer stage at the approximate focal point of the HIFU transducer (63 mm).
2. Connect the HIFU transducer (geometric focus 63 mm) to the impedance matching circuit, then power amplifier (as shown in Figure 1). Then connect the membrane hydrophone directly to the data acquisition system, ensuring that a trigger signal is provided from the function generator connected to the power amplifier (Figure 1).
3. Set the output voltage of the function generator to 30 mV, with a 10 cycle 3.3 MHz sine wave at a pulse repetition frequency of 100 Hz.
4. Using the measurement software (see **Table of Materials**) to visualize the detected acoustic signal and the micrometer stage, position the detected acoustic pulse at the correct time of flight (42.5 µm). Using only a single radial direction at a time on the micrometer stage, maximize the detected acoustic signal. Once confident this has been achieved, close the software and leave the membrane hydrophone in its current position.
5. Vary the output voltage of the function generator from 20-400 mV in 20 mV increments. At each voltage level and using the MatLab acquisition software, record the hydrophone signals. Acquire 100 pulses at each level and convert from voltage data in pressure using the provided calibration data. Average the data and measure both the peak positive and negative values for all output voltage levels. This gives the calibration data for the free field peak negative pressure to be used for both the pulse and continues wave studies.

### 3. Configuring Experimental Apparatus for Both Pulsed and Continuous Wave Studies

1. Fill an acrylic water tank (280x141x132 mm) with 4.5 L of deionized and degassed water. Mount the HIFU transducer and the co-aligned broadband hydrophone onto a three-axis manual micrometer stage. Then, fully submerge the transducer and hydrophone in the water tank. A schematic of this is shown in Figure 1.
2. Connect the HIFU transducer to an impedance matching circuit, to enable it to be driven at its third harmonic (3.3 MHz). This circuit is connected directly to the output of a RF power amplifier. A digital function generator is connected to the input of the power amplifier, and programmed remotely.
3. Prior to exposures in phantom material, use a calibrated differential membrane hydrophone to measure the peak negative pressure generated from this system for a given input voltage on the function generator as described in 2. Use these reference voltage values to set the required pressure level on the digital function generator.
4. Connect the broadband hydrophone (geometric focus 63 mm) that is housed in the central aperture of the HIFU transducer directly to a 5 MHz high pass filter. Then connect it to a 14-bit data acquisition card (DAQ) via a 40 dB preamplifier. Ensure that the high pass filter is connected with the correct bias. NOTE: This card was installed in a desktop PC and is used to control all hardware (examples this software can be found as supplementary files) and save data for off-line processing during this study.
5. Connect a transistor-transistor logic (TTL) digital delay pulse generator with Bayonet Neill-Concelman (BNC) cables to both the pulsed laser system and function generator to ensure synchronization between these systems, which will ensure that the 7 ns laser pulse is coincident in the target region during the fourth rarefaction peak from the HIFU transducer.
6. Using the method described in 1, omit the BSA and nanoparticles to make an alignment phantom, which is standard phantom material that contains a 1 mm spherical metallic target (a ball bearing). In order to achieve this, pour 25 mL of phantom material into a mold and add 62.5 µl TEMED catalyst, then wait approximately 20 min to set. Then place the metallic target centrally in the phantom and add a further 25 mL of phantom solution followed by the 62.5 µl TEMED catalyst and a further 20 min wait.
7. Place the alignment phantom into the 3-D printed holder^6^, mount on an automated 3-D stage, and approximately position so that the metallic target is at the focal peak of the HIFU transducer.
8. Using the HIFU transducer to send out a short duration 10 cycle burst (3 µs) and the hydrophone to receive (connected directly to the DAQ card), the position relative to the alignment target is optimized through pulse-echo location. The real time detected signal will be displayed on the computer. Adjust the time of flight and signal amplitude using the manual micrometer stage that the HIFU transducer and hydrophone is mounted on. Once the time of flight is set to 85 µs (a single round trip) and the signal amplitude has been maximized in both radial directions, this system will be aligned.
9. Couple the optical energy from the optical parametric oscillator (OPO) pumped by the 532 nm nanosecond pulsed laser into the phantom using a 2 mm fiber bundle. Mount this fiber onto a second micrometer stage and position at an angle of 45˚ from the acoustic axis in front of the phantom (Figure 1). The wavelength of the laser light is set to 680 nm to be visible for alignment. Once visible, position the laser illumination with the micrometer stage such that the alignment target is central in a 15 mm diameter laser spot.
10. Position the 20-90x digital microscope (working distance 90 mm) and a white light source on opposite sides of the water tank perpendicular to the propagation plane of the HIFU transducer. The microscope is mounted on a small micrometer stage. Position it such that the metallic alignment targeted is central and in focus in its field of view (5x6 mm). NOTE: After the above procedure is completed, all elements of this system (HIFU transducer, hydrophone, laser illumination and microscope) are now co-aligned to a specific location. The alignment phantom can now be replaced with the tissue-mimicking phantoms used for the study. As the phantom is mounted in a holder attached to a 3-D positioning system, different regions can be targeted whilst maintaining alignment.

### 4. Cavitation Threshold Detection from Pulsed HIFU Exposures

NOTE: The following procedure is the same for phantoms with or without nanoparticles, and should be repeated three times.

1. Ensure that the PCD system is connected after being disconnected for the alignment procedure outlined in 3.8 and tune the laser wavelength to the SPR of the nanoparticles. Using a custom control program, set the function generator to produce a 10 cycle (3 µs) HIFU burst, which is synchronized with the laser system. Also use this program to set a laser fluence of 0.4, 1.1, 2.1, or 3.4 mJ/cm^2^ though changing the timing between the triggering of the flash lamp firing and Q-switch opening in the laser system.
2. Target the focal peak of the HIFU system 10 mm deep into the phantom, and at 13 unique locations, spaced by 5 mm, in the vertical direction. In each of these locations perform an exposure at a single peak negative HIFU pressure, with the four laser fluences stated in 4.2.
3. Use the range of peak negative pressures 0, 0.91, 1.19, 1.43, 1.69, 1.92, 2.13, 2.34, 2.53, 2.71, 2.83, 3.00 and 3.19 MPa for the following exposure conditions: laser on in a nanoparticle free phantom, laser off in a nanoparticle phantom, and laser on in a nanoparticle phantom. To simulate a 'sham' laser exposure, run the system as described, but shut the manual shutter on the output of the OPO. This approach will ensure that any RF noise generated will still be present to the PCD system.
4. Program all settings and exposure positions into the control program, then execute to perform these measurements. PCD data is digitized and stored directly using the data acquisition card for post-processing. For each exposure parameter, 500 repeat exposures are acquired^6^.
5. Process the broadband emissions detected by the PCD system from the short duration HIFU exposures into the phantoms using the technique detailed by McLaughlan* et al. *(2017)^6^.

### 5. Thermal Denaturation from Continuous Wave HIFU Exposures

NOTE: The following procedure is the same for phantoms with or without nanoparticles and were repeated three times.

1. Set the laser system to give a fluence of 3.4 mJ/cm^2^ and the function generator to give a CW exposure (every 330,000-cycle burst is synced to a laser pulse). In 11 unique locations in the phantom, select a peak negative pressure of 0.20, 0.62, 0.91, 1.19, 1.43, 1.69, 1.92, 2.13, 2.34, 2.53 or 2.71 MPa.
2. Use a total exposure time of 17 s in order to acquire 1s of baseline before and after a 15 s CW HIFU exposure in the phantom. During this total exposure time, the data acquisition system is recording the PCD data. The microscope is connected to the control PC and the image frames are recorded during this time to provide a direct visualization of thermal lesion formation.
3. Repeat the process in 4.3 for all the different exposure conditions outlined in 4.4.
4. Process all PCD data off-line to calculate the inertial cavitation dose^25^ for each exposure.

## Representative Results

### Cavitation detection from pulsed HIFU exposures

The passive cavitation detection system recorded the voltage/time data for the range of HIFU and laser exposures in both phantoms with and without nanoparticles. Figure 2 shows the representative results for a range of exposures. The time scales on these plots are truncated to highlight the regions where broadband acoustic emissions would be expected, due to the time of flight of these emissions. Figure 2 demonstrates that it is only when there is a combination of nanoparticles, HIFU exposure and laser illumination that broadband emissions are detected. However, this is still a threshold phenomenon, as at the lower acoustic pressure for Figure 2h broadband emissions were not detected. The duration of these emissions typically correspond to the length of the HIFU exposure, which was around 10 µs in this study.

### Thermal denaturation from a CW HIFU exposure

Figure 3 shows a series of frames acquired from the universal serial bus (USB) camera during a single HIFU exposure with laser illumination, for the three different exposures types (with/without laser illumination and/or nanoparticles). This figure shows an example of the formation of thermal lesions in the gel phantoms for each of these conditions. In this view the HIFU exposure occurs from left to right. For the example shown in Figure 3 the peak negative pressure was 2.53 MPa, which was the upper edge of what was used in this study.

### Recording inertial cavitation dose (ICD) from CW HIFU exposures

Figure 4 shows representative results from the calculation of ICD recorded during CW HIFU exposures. This data was post processed from the emissions recorded by the PCD system during the exposure. **Figures 4a, 4c, **and** 4e** show that at a lower peak negative pressure, no broadband emissions were detected, where **Figures 4b, d, **and** f** show that ICD was recorded throughout the exposure. The highest ICD signals were observed during the exposure in a gel containing nanoparticles with both HIFU and laser exposures (Figure 4f).

**Figure:**
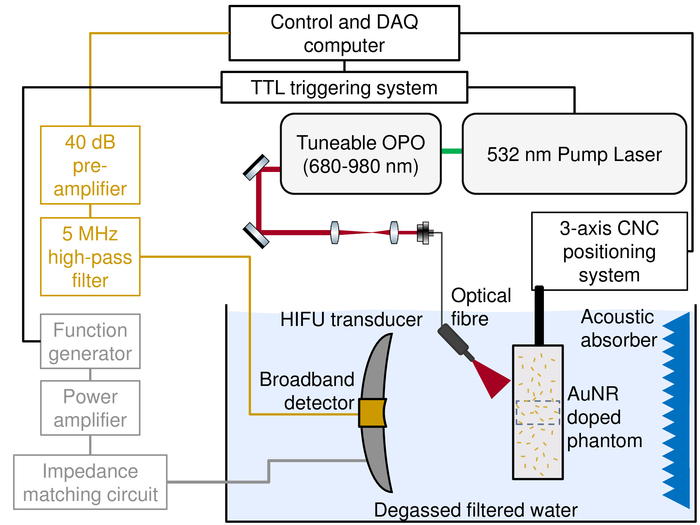

**Figure 1. A schematic representation of the experimental apparatus used in this study.** For clarity, the USB microscope and light source are omitted, but the view region is illustrated by a blue dashed box. CNC - Computer numerical control, AuNR - Gold nanorods. Figure adapted from McLaughlan* et al. *(2017)^6^. Please click here to view a larger version of this figure.

**Figure:**
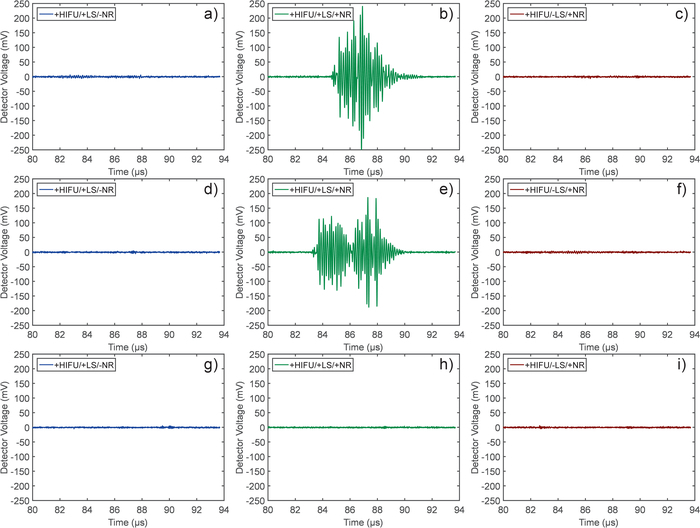

**Figure 2. An example of the voltage traces recorded with the passive cavitation detection system during short HIFU exposures, with/without simultaneous laser illumination.** When used, the laser fluence was 2.1 mJ/cm^2^ with a peak negative pressure of (a-c) 3.0, (d-f) 2.13 and (g-i) 1.43 MPa. LS - Laser, NR - nanoparticles. Please click here to view a larger version of this figure.

**Figure:**
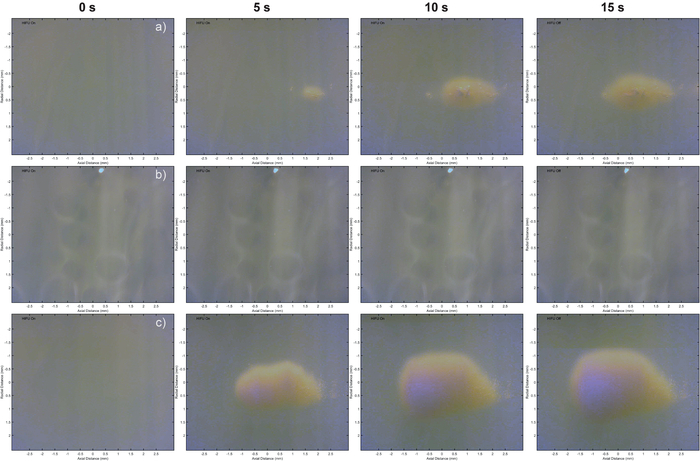

**Figure 3. Individual frames at times 0, 5, 10 and 15 s during a HIFU exposure recorded by the USB microscope.** The laser fluence was 3.4 mJ/cm^2^ and peak negative pressure of 2.53 MPa. Sequence (a) was with the laser exposure and in a phantom without nanoparticles, (b) is without laser exposure and in a phantom containing nanoparticles, and (c) has both laser illumination and a phantom containing nanoparticles. Please click here to view a larger version of this figure.

**Figure:**
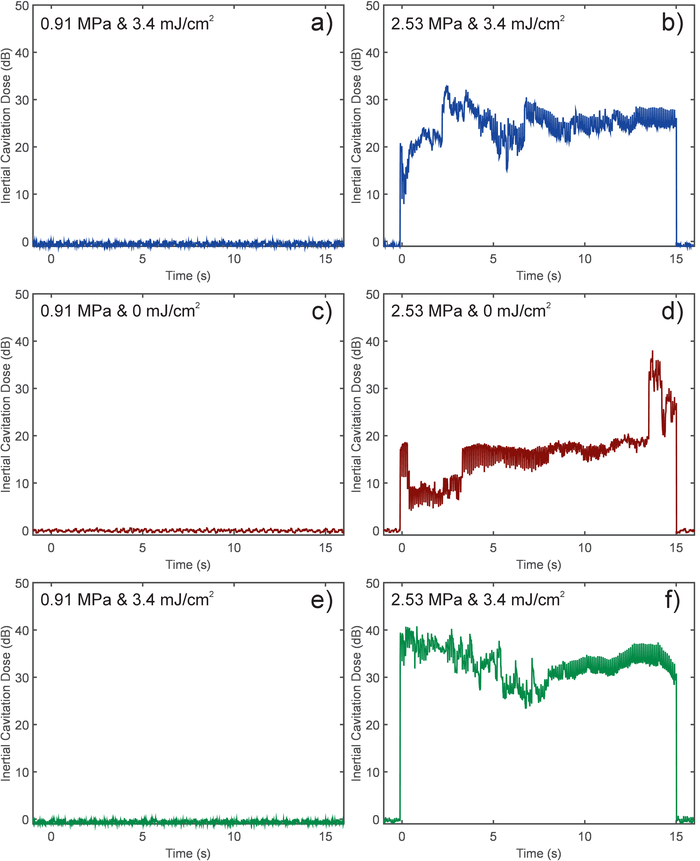

**Figure 4. Calculated inertial cavitation dose (ICD) recorded during exposures (a, b, e, & f) with and (c & d) without laser illumination.** Peak negative pressure was either (a, c, & e) 0.91 or (b, d, & f) 2.53 MPa. The phantom used in (a & b) did not contain any nanoparticles. Please click here to view a larger version of this figure.

## Discussion

This protocol is divided into four separate sections, describing the manufacture of the tissue-mimicking phantom through to the CW exposures in them to produce thermally generated denaturation. This denaturation of the phantoms simulates thermally generated coagulation necrosis experienced by soft tissue exposed to HIFU^1^. In their manufacture, it is important to ensure that the ratio of APS and TEMED is such that the process does not catalyze too quickly. As this process is exothermic, the faster this rate, the higher the temperature reached^25^ and thus could denature the BSA proteins prior to exposure. The ratio of APS to TEMED in this protocol has been set such that this should not occur, however the molds could be placed in ice water during the polymerizing of the gel to further minimize this possibility.

As this protocol focuses on the nucleation of cavitation through combining nanoparticles, laser illuminations and HIFU exposure, a critical step in the manufacture of the gel phantoms is to degas them under vacuum for a minimum of 30 min. Once exposed to HIFU (particularly CW exposures), even if a thermal lesion was not present, it is important to target a fresh location in the gel phantoms to avoid preexisting nuclei. When moving the phantom using the computer controlled translation system it is important to ensure that the depth of the HIFU focus (and thus aligned region) is kept consistent. This ensures that the HIFU pressure and laser fluence levels are uniform for each specific exposure parameter. For this protocol and after the initial placement of the phantom holder, it is then only translated in the vertical axis.

The temperature-sensitive tissue-mimicking gels are used widely by the HIFU research community^25^, as they provide a visual mechanism for monitoring the formation of a thermal lesion. This study was the first example of combining them with nanoparticles and demonstrating the enhancement provided to lesion formation through controlled cavitation activity. However, although they are classified as tissue-mimicking for their response to temperature, both their optical and acoustic attenuation are not. Due to the need to visualize the lesion formation in the gels, the phantoms are near transparent, with a slight yellow tint. As the laser fluence is adjusted to account for this, it does mean that the laser light illuminating the target region is collimated rather than diffusive as would be for normal tissue. Thus to allow for clinical translation multiple illumination sources would be needed to ensure enough fluence on the surface. Currently this work adheres to the guidelines^22^ for the safe use of lasers when exposed to skin. This would limit the maximum laser fluence achievable at depth; thus, this technique would initially be suited to treating superficial cancers such as breast, or head and neck. Furthermore, plasmonic nanoparticles targeted to surface receptors for these types of cancers could provide increased selectivity in treatments. However, even though this is a highly active area of research, no such particles are currently approved for clinical use.

The acoustic attenuation of the phantoms with nanoparticles was measured to be 0.7±0.2 dB/cm^6^, and, compared with the value for soft tissue of 3-4 dB/cm, it is significantly lower. Thus, the heating from HIFU exposures in these gels would be lower than would be observed in soft tissue. It has been demonstrated that addition of glass beads to the gel increases the attenuation levels similar to soft tissue^25^. However, in this application, this approach is not possible as these beads would act a nucleation sources for cavitation activity even in the absence of nanoparticles, and thus misrepresent the cavitation threshold. When comparing the heating efficiency for with the results from the study by Choi* et al. *(2013)^25^, thermal lesions were generated at peak pressure ranges of 14 - 23 MPa (it is not stated if this was peak positive or negative pressure). As this was performed at 1.1 MHz, the attenuation in the phantoms was lower than used in this study. Nevertheless, the nanoparticle-nucleated approach in this study was able to generate thermal lesions in these phantoms at pressures ranging from 1.19 to 3.19 MPa, thus demonstrating an increased efficiency over current methodologies.

Future testing for this methodology should be undertaken in an *in vivo *model to incorporate tumor reduction, tissue perfusion, molecular targeting of nanoparticles and relevant acoustic attenuation parameters.

## Disclosures

The author has nothing to disclose.
